# Stochastic Simulations of Clusters: Quantum Methods in Flat and Curved Spaces. By Emanuele Curotto, CRC Press, 2010; 696 pages, Hard Cover. Price: $159.95/CHF 257.00 ISBN 978-1-4200-8225-8

**DOI:** 10.3390/ijms10104435

**Published:** 2009-11-20

The following paragraphs are reproduced from the website of the publisher [1] or out of *Stochastic Simulations of Clusters; Quantum Methods in Flat and Curved Spaces* [2].

Clusters hold the key to our understanding of intermolecular forces and how these affect the physical properties of bulk condensed matter. They can be found in a multitude of important applications, including novel fuel materials, atmospheric chemistry, semiconductors, nanotechnology, and computational biology. Focusing on the class of weakly bound substances known as van derWaals clusters or complexes, *Stochastic Simulations of Clusters: Quantum Methods in Flat and Curved Spaces* presents advanced quantum simulation techniques for condensed matter.

The book develops finite temperature statistical simulation tools and real-time algorithms for the exact solution of the Schrödinger equation. It draws on potential energy models to gain insight into the behavior of minima and transition states. Using Monte Carlo methods as well as ground state variational and diffusion Monte Carlo (DMC) simulations, the author explains how to obtain temperature and quantum effects. He also shows how the path integral approach enables the study of quantum effects at finite temperatures.

To overcome timescale problems, this book supplies efficient and accurate methods, such as diagonalization techniques, differential geometry, the path integral method in statistical mechanics, and the DMC approach. Gleaning valuable information from recent research in this area, it presents special techniques for accelerating the convergence of quantum Monte Carlo methods [1].

Features [2]:

- Covers a broad scope of quantum simulation aspects, from FORTRAN programming through the classical physical laws and the algorithms for their integration to the fundamentals of statistical mechanics.
- Applies linear algebra, Lie algebra, and differential geometry to computations
- Explains how to perform state-of-the-art vector space quantum mechanics calculations, such as the discrete variable representation coupled with the Lanczos algorithm
- Introduces real and imaginary time evolution methods in quantum mechanics
- Presents applications to atomic clusters
- Shows how to carry out DMC and path integral simulations in curved spaces, which have proven crucial for simulating molecular clusters.
- Provides simulations in nonrelativistic wave mechanics and statistical thermodynamics

Table of Contents [2]:

- Fundamentals
  1. FORTRAN Essentials
  2. Basics of Classical Dynamics
  3. Basics of Stochastic Computations
  4. Vector Spaces, Groups and Algebras
  5. Matrix Quantum Mechanics
  6. Time Evolution in Quantum Mechanics
  7. The Path Integral in Euclidean Spaces
- Atomic Clusters
  1. Characterization of the Potential of Ar_7_
  2. Classical and Quantum Simulations of Ar_7_
- Methods in Curved Space
- Introduction to Differential Geometry
- Simulations in Curved Manifolds
- Applications to Molecular Systems
  1. Clusters of Rigid Tops

## Footnotes

1. The website for this book is http://www.crcpress.com/product/isbn/9781420082258/.

2. Curotto, E. *Stochastic Simulations of Clusters: Quantum Methods in Flat and Curved Space*, 1st ed.; CRC Press, Taylor & Francis Group: Boca Raton, FL 33487-2742, USA, 2010.

